# Pediatric Obstructive Sleep Apnea and the Critical Role of Oral-Facial Growth: Evidences

**DOI:** 10.3389/fneur.2012.00184

**Published:** 2013-01-22

**Authors:** Yu-Shu Huang, Christian Guilleminault

**Affiliations:** ^1^Department of Child Psychiatry and Sleep Center, Chang Gung Memorial Hospital and UniversityTaiwan, China; ^2^Sleep Medicine Division, Stanford UniversityRedwood City, CA, USA

**Keywords:** pediatric sleep-disordered-breathing, non-obese, oral-facial anatomy, hypotonia, oral-facial growth, oral-facial myofunctional dysfunction

## Abstract

**Aims:** Review of evidence in support of an oral-facial growth impairment in the development of pediatric sleep apnea in non-obese children. **Method:** Review of experimental data from infant monkeys with experimentally induced nasal resistance. Review of early historical data in the orthodontic literature indicating the abnormal oral-facial development associated with mouth breathing and nasal resistance. Review of the progressive demonstration of sleep-disordered-breathing (SDB) in children who underwent incomplete treatment of OSA with adenotonsillectomy, and demonstration of abnormal oral-facial anatomy that must often be treated in order for the resolution of OSA. Review of data of long-term recurrence of OSA and indication of oral-facial myofunctional dysfunction in association with the recurrence of OSA. **Results:** Presentation of prospective data on premature infants and SDB-treated children, supporting the concept of oral-facial hypotonia. Presentation of evidence supporting hypotonia as a primary element in the development of oral-facial anatomic abnormalities leading to abnormal breathing during sleep. Continuous interaction between oral-facial muscle tone, maxillary-mandibular growth and development of SDB. Role of myofunctional reeducation with orthodontics and elimination of upper airway soft tissue in the treatment of non-obese SDB children. **Conclusion:** Pediatric OSA in non-obese children is a disorder of oral-facial growth.

## Introduction

Since obstructive sleep apnea syndrome (OSAS) first was reported in children in Guilleminault et al. (1976), recognition of abnormal breathing during sleep has progressed. Prior to the introduction of the nasal cannula-pressure transducer (Serebrisky et al., 2002), thermistors were used to score abnormal breathing during sleep in association with esophageal manometry (Pes). The nasal cannula-pressure transducer is more accurate than its predecessor, and it allows for recognition of the “flow limitation” breathing pattern. This pattern is associated with an abnormal increase or decrease in respiratory effort associated with EEG changes that occur during sleep disturbances (Hosselet et al., 1998; Aittokallio et al., 2001; Lin and Guilleminault, 2011). These sleep EEG changes were also shown to be better recognized using the “cyclic alternating pattern” (CAP) scoring system, a visual scoring system commonly used in Europe and Latin America (Terzano et al., 2002). This visual scoring system recognizes sleep disturbances, particularly arousals indicative of sleep disruption, better than the most commonly used atlas, which requires disturbances to occur for at least 3 s to be scored. More accurate approaches have been used, such as computerized analyses of the sleep EEG based on specific algorithms (Chervin et al., 2004) or using well-known EEG analysis programs (e.g., fast-Fourier Transform, Wavelet, and Hiller–Huang Transform programs). Usage of these recording techniques has improved recognition of Sleep-Disordered-Breathing (SDB).

Poor tolerance of early cases of children treated with tracheostomy and home nasal CPAP (Sullivan et al., 1981) led to the advent of maxillomandibular advancement (MMA) surgery as a procedure designed to target more specifically the upper airway (Powell et al., 1983). Follow-up of one case for more than 25 years post-MMA demonstrated lasting and complete resolution of OSAS.

## Lessons from OSA Treatment with Adenotonsillectomy

Despite the widespread use of limited techniques to identify the complete cessation of abnormal breathing and its effects during sleep, many studies have demonstrated significant improvement in SDB without complete elimination of the phenomenon. Two studies showed that prepubertal adolescents initially considered to have been cured by adenotonsillectomy subsequently had recurrence of OSA as teenagers (Guilleminault et al., 1989; Tasker et al., 2002). In Guilleminault et al. (1989), subjects had narrowing behind the base of the tongue and oral-facial anatomical abnormalities that either did not exist initially or had not been identified previously. Tasker et al. (2002) also confirmed the presence of abnormal upper airway anatomy and SDB in subjects 12 years after adenotonsillectomy. This phenomenon was observed again in more recent larger studies. Guilleminault et al. (2004) demonstrated complete resolution of OSA following adenotonsillectomy in only 51% of non-obese prepubertal children that were studied with polysomnogram (PSG) 3 months post-operatively, an observation confirmed in later studies (Tauman et al., 2006; Guilleminault et al., 2007). In a more recent multi-center study (Bhattacharjee et al., 2010) with 500 subjects, half of whom were obese, adenotonsillectomy again led to improved clinical symptoms and PSG results but was not curative in 70% of the cases. Chen et al. (2012) conducted a prospective study of prepubertal children treated with adenotonsillectomy with subsequent normal PSG testing as defined by AASM 2007 scoring criteria (Iber et al., 2007). The prospective 5-year follow-up study included systematic PSG testing, clinical evaluations, attentional neurocognitive testing, and 3D-CT of the upper airway. The follow-up evaluations performed at 3 and 6 months showed normal PSG and neurocognitive scores. However, further follow-up once again demonstrated the simultaneous presence of abnormal breathing during sleep (AASM criteria) and abnormal neurocognitive test results in 40% of the subjects. Moreover, 3D-CT analysis showed abnormal oral-facial development in these children.

Kim and Guilleminault (2011) looked at the presentation of anatomical oropharyngeal abnormalities in 400 prepubertal children diagnosed with OSA who had enlarged adenotonsillectomy before otolaryngological treatment (Friedman et al., 1999). Nearly all of the children with OSA had at least one type of oropharyngeal abnormality from a list of pre-defined potential problems. Consistent with prior studies, clinical symptoms of OSA and abnormal PSG results persisted in some children at 3-month post-operative follow-up. However, presence of a pre-defined oropharyngeal abnormality was scored and was insufficient to predict post-surgical outcome on OSA.

Further analyses defined more specific clinical examination findings that better predicted persistence of abnormal post-adenotonsillectomy PSG results. These included the presence of a Mallampati scale score of 3 or 4 (Mallampati et al., 1985; Guilleminault et al., 2007), the presence of a deviated nasal septum, and the presence of a small mandible. The Mallampati scale does not identify one particular anatomical abnormality, but rather it represents a combination of deficiencies involving both the nasomaxillary complex and the position of the mandible. These findings suggest the need to assess anatomic elements of the upper airway in terms of oral-facial growth and impairment of general oropharyngeal growth rather than merely tallying the quantity of anatomic abnormalities.

## Lessons from Orthodontia and the Experimental Infant Monkey Model

European orthodontists showed that abnormal nasal resistance induced by enlarged adenoids and tonsils in children were associated with mouth breathing and led to important craniofacial changes (Haas, 1961; Linder-Aronson, 1969, 1970; Wertz, 1970; Timms, 1974, 1984; Gray, 1975; Hershey et al., 1976; McNamara, 1981; Löfstrand-Tideström et al., 1999; Pirila-Parkkinen et al., 2009). Adenotonsillar ablation led to cessation of mouth breathing and progressive restoration of normal facial development facilitated by orthodontia use.

Other orthodontists, concerned by the negative impact that a narrow maxilla imparts on teeth positioning and facial growth during prepubertal development, performed “rapid maxillary expansion” (RME) and reported that such treatment also had made an impact on sleep-related complaints. In one study, children treated with RME experienced elimination of nocturnal enuresis (Timms, 1974).

However, the most important findings were obtained on infant rhesus monkeys, when the important role of abnormal nasal resistance during the developmental period was demonstrated. Between 1970 and 1980, a number of very important experiments on newborn rhesus monkeys were performed, whereby a small silicone head was placed within the nostrils of infant monkeys and held in place by a thin thread in order to induce nasal resistance for the first 6 months of life (Harvold et al., 1981; Vargervik et al., 1984). The blockade of the nasal passage led to narrowing of dental arches, decrease in maxillary arch length, anterior cross bite, maxillary overjet and increase in anterior facial height (Harvold et al., 1981). Experimentally induced abnormal nasal resistance led to systematic changes in the oral-facial muscles. The changes were noted in the systematic recording of different muscles, in particular the geniohyoid, the genioglossal muscles of the tongue, the suprahyoid dorsal tongue fibers, the upper lip elevators, and the digastric muscles. EMG testing showed abrupt induction of rhythmic discharge patterns, a stark contradiction to the nearly continuous and desynchronized discharges in most normal subjects. Tonic EMG discharges changed back to the normal pattern when nasal breathing was restored at the end of the 6-month experiment (Vargervik et al., 1984 and Miller et al., 1984).

Increased nasal resistance has a dramatic effect on the maxillomandibular skeleton, halting growth (Harvold et al., 1981), and bringing about adaptive changes in the soft tissues that are associated with deviation in jaw posture and tongue activity (Miller et al., 1984; Vargervik et al., 1984). Obstruction of nasal airflow induces functional changes in the nasomaxillary complex and on the mandible. In the subject group of newborn rhesus monkeys, there were several consequences: an absence of development, which impacted the maxilla and restricted the nose and upper jaw; displacement of the mandible leading to mouth breathing; and oral breathing that developed in association with increased nasal resistance, leading to mouth opening and mouth breathing that occurred in the awake and sleep states. These changes led to the narrowing of the cranial skeleton (Harvold et al., 1981; Miller et al., 1984; Vargervik et al., 1984; Rubin, 1987; Vargervik and Harvold, 1987). These changes were shown to be reversible if the experimental nasal resistance was withdrawn while the infant monkey was still in its developmental phase.

These experiments taught us that in growing animals in which the nasal airway is gradually occluded, there is an adverse effect on the morphology of the nasomaxillary complex, mandible, and pharyngeal airway space. The morphometric changes are induced by altered functioning of the muscles with changes in muscle firing that are triggered by abnormal nasal resistance. Unfortunately, OSAS largely was unknown at the time of these investigations and no sleep recordings were performed on the subject animals.

## Application of Work in Orthodontia in the Field of SDB

More recent investigations demonstrating incomplete resolution of abnormal oropharyngeal growth by adenotonsillectomy have led to the usage of orthodontic techniques to help treat pediatric SDB.

Based on prior research demonstrating the important role of the mesio-palatine suture in the nasomaxillary complex growth, much investigative effort has been invested in examining the complex’s ossification process. Cartilage is a connective tissue made of chondrocytes embedded in a collagen-rich matrix (particularly type II collagen), associated with proteoglycans in hyaline cartilage that strengthens it, as well as elastin (depending on the type of cartilage). Hyaline cartilage is the forerunner to skeletal bones in the fetus, and endochondral ossification is the process leading to formation of the nasomaxillary complex.

Rapid Maxillary Expansion (Pirelli et al., 2004) is a procedure applying orthopedic forces on the mid-palatal sutures using the first molars and permanent premolars as anchor teeth. While in deciduous dentition, the second primary molars are selected as long as they can provide the required firmness. The device is composed of a central expansion screw with four arms: two front arms and two back arms. The bone distraction (enlargement) at the suture level enables an effective enlargement of the maxillary skeletal base. Enlargement is visually appreciable with X-ray (as the gain appears as a radiotransparency corresponding to the visually seen space) as the bone distraction leads to an interincisive space (a diastema). The procedure usually takes 3–4 weeks with daily turning of a midline screw that allows distraction of the space at the level of midline suture. The transpalatal force, which exceeds the orthodontic one, produces an orthopedic force that opens the mid-palatal suture leading to maxillary movement without tipping teeth. Once the needed extension is obtained (end of the activation phase), the midline screw is locked and the device is kept in place for at least 4–6 months. This time period allows the newly formed bone to strengthen. However, this does not generate cartilage in the mandible. Nevertheless, manipulation and verticalization of teeth can stimulate mandibular growth and such bimaxillary distraction is often needed in OSA children. In addition, maxillary widening also seems to impact mandibular growth independently.

Contrary to its efficacy in lateral expansion, RME is limited in anteroposterior lengthening capabilities. In the past, appliances such as the Herbs appliance or its equivalent were thought to be capable of producing anterior-posterior growth in prepubertal children. However, while such appliances may protrude the lower jaw forward, there is no evidence currently that more growth than expected with age is attained. Distraction osteogenesis may be performed in these cases, but while such an approach is performed in children with clear malformations at birth, it has not been recommended in non-syndromic children with OSA until oral-facial growth is well advanced (Guilleminault and Li, 2004).

In normal individuals, 60% of facial growth is attained by 6 years and about 90% by 11–12 years of age. Thus, distraction osteogenesis is not usually performed before approximately 14 years of age in non-syndromic children with OSA. Even then, it must be determined whether the anteroposterior advancement will be sufficient on its own or the teenager will need both anteroposterior and lateral extension. If it is the latter scenario, as is most commonly the case, MMA (Holty and Guilleminault, 2010) is the best option. On the other hand, distraction osteogenesis may be useful in certain cases, such as in the elimination of residual OSA.

In summary, several studies have shown that RME or bimaxillary distraction have a clear impact on pediatric OSA and may resolve the residual symptomatology seen in post-adenotonsillectomy patients. The combination of adenotonsillectomy and RME leads to complete resolution of OSA symptoms in some cases, and a small prospective follow-up study demonstrated sustained results 36 months post treatment (Villa et al., 2011).

Two investigations have looked at the effects of RME versus adenotonsillectomy. In the first study, subjects presented with narrow jaws and both adenoid and tonsillar enlargement (3+ on the Friedman scale). Assignment to the initial treatment groups of RME and adenotonsillectomy was randomized. With the exception of one child who improved with orthodontic treatment alone, all subjects required both adenotonsillectomy and orthodontic treatment to see improvement (Guilleminault et al., 2011). In the second study, children with infectious tonsils were treated with adenotonsillectomy while the others were designated to the orthodontic treatment group, with the design to send the patients into the other treatment arm if initial therapy yielded incomplete results. In this study, more children were treated only with orthodontics, indicating that oral-facial factors may be dominant in at least a subgroup of OSA children (Pirelli et al., 2012). In both studies, several children were not completely cured with these approaches, indicating that more aggressive treatment may be needed. Persistent oral-facial problems were always identified as the prominent factor associated with failure to achieve a complete cure of OSA.

These investigations demonstrate that adenotonsillectomy in non-obese children does not cure OSA in many prepubertal children, and that oral-facial anatomical problems play a pivotal role in the development of OSA in children. Moreover, for some subjects these anatomical problems may be amenable to orthodontic treatment.

## Interaction between Adenotonsils and Oral-Facial Growth and Evidences from Prematures Infants

Swedish investigators suggested that children first become mouth breathers, and the subsequent subjection to repetitive abnormal stimulations resulting from mouth breathing causes an inflammatory reaction in the tonsils (Zettergreen et al., 2002). The resulting tonsilar enlargement involves inflammatory factors, such as leukotriene.

In Taipei (Taiwan), YS Huang has created a prospective cohort of 300 infants born between 25 and 37 weeks of gestational age. These infants are evaluated within 1 week following birth, then at 3, 6, 12, 18, 24, and 36 months of age. These children were evaluated for clinical development and neurologic function, including feeding behaviors, actigraphy, PSG, and systematic photographs of the face (frontal and lateral) and oral regions. Fiber optic illumination was used in the photographs of the oral regions to evaluate the size and presentation of the hard palate, and these photos were scored blindly by a specialist uninvolved in the clinical evaluations.

### Preliminary results

Three hundred children involved in the cohort have been followed until at least 24 months of age. At this time, infants who had nasal or mouth tube placed at birth were eliminated from evaluation. All infants born below 34 weeks of gestational age were found to have high and narrow hard palates (with palatal width smaller than 24 mm at 6 months post delivery). Infants born at 37 weeks had palates whose widths measured between 27 and 31 mm, while in 35% of infants born at 36 weeks and in all infants born sooner, they measured less than 27 mm. The measurements correlated directly with gestational age.

Problems with feeding behavior were present in infants born at 36 weeks and younger. All infants with extended in-hospital stays due to premature birth were bottle fed, while term infants were breast fed. All infants held in the hospital postnatally were separated from their mothers and bottle fed either with formula or expressed breast milk.

Currently 82% of the studied infants (*n* = 207/252) have presented with a high and narrow hard palate, as well as apnea and/or hypopnea during sleep. Mouth breathing has been noted in these children and has become more apparent with age, as noted during the first follow-up visit at 3 months post-partum.

All children born at 35 weeks or earlier exhibited limb hypotonia during the general neurological evaluation. Some older infants also demonstrated hypotonia, but the frequency of this was inversely proportional to pregnancy duration. Hypotonia was defined by the persistence of a positive “scarf” sign (i.e., position of elbow, crossing the midline, when arm is pulled toward opposite side), which was noted in infants born as late as 37 weeks (Korobkin and Guilleminault, 1979). At 6 months old, the 207 subjects without history of intubation showed no signs of abnormally enlarged tonsils, but all of them presented with a high and narrow hard palate.

Enlarged tonsils when present were only noted in later examinations in children who exhibited mouth breathing, high and narrow hard palates, and mouth breathing during sleep. The hypothesis that the enlargement of tonsils occurs as a result of mouth breathing and the presence of a high and narrow hard palate is supported here. The effect on adenoids was not observed in this study, since otoscopy of the back of the nose was not performed. Nevertheless, documentation of a high and narrow hard palate at birth predicts the presence of abnormal oral-facial features existing from birth in most cases.

## Development of Abnormal Hard Palate after Birth

The most interesting cases are the small fraction of subjects (9%, *n* = 23), that had a normal hard palate at birth, but then developed an abnormal hard palate by the 6-month-old follow-up visit. All of these children were in the 36 weeks and older gestational age group. None of them had ICU hospitalizations, but they did show positive scarf signs at 3 months follow-up evaluation. Also, all of these children were bottle fed due to difficulties with breast feeding. At the 6-month evaluation, their tongues were flat and low lying as observed by examination and photography, a presentation similar to that of a hypotonic tongue. These children had normal breathing during sleep at birth evaluation, but developed SDB as documented by sleep recordings during the follow-up period.

## Infants with Normal Palate at Follow-Up

In this study only 9% of subjects (*n* = 22) had a completely normal hard palate, normal breathing during sleep, and normal development. With the exception of a pair of twins who were born at 34 weeks, all were in the 36 weeks and older age group and had normal breast feeding. The twins were followed by a special myofunctional reeducation team applying tongue reeducation techniques to strengthen the tongue and oral muscles in the early postnatal period (Page, 2003; Bahr, 2010). They were bottle fed with a special “hard” nipple, with the hardness and size adjusted overtime to elicit more effort from their tongue muscles when feeding.

## Role of Oral-Facial Muscle Hypotonia and Usage of Myofunctional Reeducation

The investigation of infant monkeys showing changes in EMG firing demonstrated that abnormal nasal resistance early in life leads to mouth breathing associated with abnormal muscle tone, oral-facial hypotonia, and secondary changes in maxillary-mandibular growth (Harvold et al., 1981; Miller et al., 1984; Vargervik et al., 1984; Vargervik and Harvold, 1987). In the 1970s, many researchers studied the many important functions oral-facial muscles played, including swallowing, breathing, phonation, mastication, facial mimicry, and overall head posture (Leech, 1958; Ricketts, 1958; Hawkins, 1965; Linder-Aronson, 1969, 1970; Solow et al., 1984; Rubin, 1987; Behlfelt et al., 1990).

Orthodontists across Europe concluded that myofunctional reeducation of the oral-facial region was an important part of treatment aimed at correcting abnormal maxillary and mandibular growth, as well as normalizing bite and teeth positioning. This was due to its effect in rehabilitating abnormal local muscle activity (Chauvois et al., 1991).

Creation of oral-facial muscle reeducation programs meant specialized re-educators had to be trained, which led to specific university training. Combined orthodontic and myofunctional reeducation was thereafter applied to children with narrow jaws. Looking at long-term outcomes, combination therapy was more successful than either treatment individually. More recently, after demonstrating the involvement of maxillary-mandibular growth problems in SDB, children were treated with both myofunctional reeducation and orthodontia (Chauvois et al., 1991; Guilleminault et al., 2012a,b; Guilleminault, 2012). In Brazil, these treatments were applied in children and adults, and a Brazilian team has published results of the combined treatment approach for adult OSA showing improvement of AHI in well established OSA patients (Guimaraes et al., 2009). Outcome reports for myofonctional reeducation in SDB children otherwise are rare.

However in the 1990s there has been evaluation of children with abnormal oral-facial development who received orthodontic treatment without sleep investigation (Chauvois et al., 1991), including results obtained from an appropriate reeducation regimen. Despite usage of combined approaches in specific geographic places for orthodontic problems, no prospective long-term study has been published in the treatment of SDB children. Studies recently have been initiated that compare outcome of adenotonsillectomy and orthodontic treatment without myofunctional treatment. We performed one study investigating the role of myofunctional therapy in association with orthodontia in children with SDB. While our own retrospective multi-center investigation was limited due to difficulty retrieving original data from the various locations, it produced evidence that the persistence of mouth breathing during sleep-related to myofacial hypotonia led to the reoccurrence of SDB (Guilleminault et al., 2012a). This recurrence in children treated appropriately with adenotonsillectomy and orthodontics was demonstrated, along with the presentation of clinical signs and symptoms and typical PSG findings. Myofunctional clinical evaluation revealed the presence of oral-facial hypotonia. These children also demonstrated mouth breathing during PSG.

This limited retrospective study (Guilleminault et al., 2012a) involved 24 early teenagers who previously had been diagnosed with SDB between ages 31/2 and 7 years and had been treated appropriately with adenotonsillectomy and orthodontia and also had been instructed to commence myofunctional reeducation. Recurrence of OSA at occurred in 13 subjects. Each of these presented with oral-facial hypotonia, mouth breathing during sleep, and reported not completing myofunctional reeducation. In contrast, the subjects with normal breathing at long-term follow-up had normal oral-facial tone, nasal breathing during sleep, and had completed myofunctional therapy. This study illustrates the potential importance of myofunctional treatment as an adjunctive treatment of SDB children, and that the presence of normal post-procedural PSG findings alone may not be sufficient to ensure long-term remission of abnormal nocturnal breathing.

Myofunctional reeducation is applied much less frequently in early infancy. The premature cohort investigation indicates that SDB is seen in very early life, and that abnormal anatomic features of structures limiting the upper airway are also present very early. In patients with these recognized abnormalities, application of myofunctional reeducation techniques may be helpful. Unfortunately, orthodontist exposure is rare in the pediatric arena, despite the pervasive knowledge of generalized hypotonia in premature infants.

Page (2003) speaks of the importance of dealing with oral-facial hypotonia and how to manage it in infancy, as it may be associated with negative facial anatomy problems later. There is data showing that the way an infant sucks on a nipple (breast or bottle) is important for the development of normal oral-facial muscle tone and the prevention of local hypotonia (Davis and Bell, 1991; Paunio et al., 1993; Ogaard et al., 1994). Breastfeeding is a complex reflex requiring considerable strength. During feeding premature infants may experience significant apnea associated with severe oxygen desaturation. Often they cannot breastfeed sufficiently at their mother’s breast, and therefore end up being bottle fed, since it requires less tongue strength and sucking effort.

In our premature infant prospective study, more than 90% of women with premature infants bottle fed their infants. Oral-facial hypotonia in premature infants has been the subject of much research. Page studied how to deal with this hypotonia. Bottle feeding may be performed with special nipples that require more effort from the oral-facial muscles, such as NUK-Gerber nipples (Ogaard et al., 1994), and oral reflexes may be triggered early in the postnatal period using finger stimulation of the lips and mouth. Progressive development of a normal palate can be attained using such approaches. (In one case, there was documentation of sustained results up to age 6, as reported by MJ Boileau, Department of Orthodontics Bordeaux University Dental School, France.) We conducted a non-randomized small study with five infants. It showed that when mothers followed feeding recommendations to use these special bottle nipples and engaged in finger stimulation of oral reflexes, a progressive normalization of abnormal palatal anatomy associated with normal breathing during sleep was observed at 24-month follow-up. This was not observed in gestational age-matched infants using regular nipples.

This was also demonstrated in the premature twins referenced earlier, leading to secondary development of normal oral-facial features and absence of SDB. Feeding was associated with a special pacifier, but reeducation of muscle hypotonia involved more participation from mothers, including stimulation of the infant’s lips by placing a finger there and using FDA-approved chewing toys for ages 6 months and up, such as ARK’s Grabbers™ chewing toys (Bahr, 2010). These studies are very limited and are similar to case reports, but they complement observations in older children who had recurrence of SDB after appropriate treatment but did not have myofunctional therapy (Guilleminault et al., 2012a).

In summary, premature infants as well as some full-term infants present with abnormal oral-facial features, particularly a high and narrow hard palate. These findings are associated with oral-facial hypotonia. Systematic follow-up to 36 months of age indicates persistence of abnormal tongue position and abnormal breathing, with presence of mouth breathing demonstrated on PSG. Information from orthodontists indicates that performing special oral-facial exercises during feeding, and chewing in the first 2 years of life may lead to correction of abnormal anatomy, resulting in repositioning of the tongue and development of a normal nasomaxillary complex and mandible. A small non-randomized study indicates that premature infants may develop normal nasomaxillary complex and mandible when a strong effort is made to induce normal oral-facial musculature. Independent of sleep studies, years of experience in orthodontia also supports the important role of myofunctional reeducation in the presence of abnormal oral-facial anatomy (Chauvois et al., 1991). In our investigations, absence of SDB is associated with normal nasal breathing during sleep, but recurrence of OSA during the teenage years is associated with mouth breathing during sleep and documentation of oral-facial hypotonia.

## Conclusion

The different data accumulated over time on SDB children and the experimental data obtained from infant monkeys years ago are indicative of a strong association between normal oral-facial muscle tone and the normal development of the nasomaxillary complex and mandible. Presence of abnormal muscle tone, either experimentally induced by creation of abnormal nasal resistance or due to premature birth, is associated with mouth breathing particularly during sleep, abnormal placement of the tongue, and either development or worsening of the oral-facial anatomy. In humans, SDB is noted in association with pathological hypotonia of the tongue muscles. In a small group of infants seen at birth with a normal hard palate, development of a high and narrow hard palate and SDB was documented in children with oral-facial hypotonia. When the high and narrow hard palate was noted at birth in these cases, hypotonia also was present, and SDB was noted. In rare cases efforts very early in life to counteract oral-muscle hypotonia and reverse the high and narrow hard palate may lead to normal development and absence of SDB at follow-up. As suggested by Swedish investigators, tonsillar enlargement appears to be a secondary phenomenon that further impacts nasal resistance. No information on adenoids had been collected in our infant studies, but was obtained in the long-term follow-up of older children with 3D-CT scans. Adenotonsillectomy often is insufficient to achieve complete and lasting resolution of breathing problems.

Understanding the continuous interaction between muscle activity of the tongue and other oral-facial muscles, as well as the development of normal anatomic structures supporting the upper airway may lead to expansion of myofunctional reeducation as a therapeutic tool. We still do not know when the interaction between the potential airway-limiting oral-facial anatomic structures and its musculature begins. Interruption of normal development with premature birth may explain the frequency of sleep-related breathing problems in premature infants. However, these events also can be seen in full-term infants, leading to negative consequences (Rambaud and Guilleminault, 2012). It is possible that the abnormality leading to oral-facial hypotonia begins *in utero*. Investigation of facial expression and movements shows that beginning in early pregnancy, the fetus exhibits regular movements of the mouth and face. For example, the most frequent movement seen during the second trimester is sucking (Kurjak et al., 2005). Abnormal pregnancy and/or impairment of these movements may impede normal muscle activity at birth.

## Conflict of Interest Statement

The authors declare that the research was conducted in the absence of any commercial or financial relationships that could be construed as a potential conflict of interest.
